# Time spent on the smartphone does not relate to manual dexterity in young adults

**DOI:** 10.1186/s12868-021-00639-y

**Published:** 2021-05-11

**Authors:** Luca Petrigna, Milda Treigienė, Ewan Thomas, Diba Mani, Simona Pajaujiene, Patrik Drid, Gioacchino Lavanco, Antonio Palma, Antonino Bianco

**Affiliations:** 1 Sport and Exercise Research Unit, Department of Psychology, Educational Science and Human Movement, University of Palermo, Via Giovanni Pascoli 6, 90144 Palermo, Italy; 2 Department of Coaching Science, Lithuanian Sports University, Kaunas, Lithuania; 3 Department of Applied Physiology and Kinesiology, University of Florida, Gainesville, FL 32608 USA; 4 Faculty of Sport and Physical Education, University of Novi Sad, Lovcenska 16, 21000 Novi Sad, Serbia

**Keywords:** Manual dexterity, Cognitive function, Mobile phone, Phone, Grooved pegboard test

## Abstract

**Background:**

The Grooved Pegboard Test (GPT) is widely adopted to evaluate manual dexterity, it presents normative data but the test is influenced by different factors. The influence of time spent on smartphones has not been considered before, for this reason, the objective of this study was to evaluate if smartphone use influences the time to complete the GPT. A total of 38 (21 women; 17 men) young adults 20.7 (1.5) years participated in the study. The time spent on the smartphones during the last seven days was recorded through the device itself and the GPT performance was measured. A correlation analysis between the time spent on the smartphone and GPT was performed while the *t*-test was adopted to evaluate gender differences.

**Results:**

No statistically significant differences were detected between men and women in the time to complete the GPT (p = 0.20) and in the time spent on the smartphone (p = 0.87). The GPT and the time spent using the smartphone were not correlated (r = 0.044, p = 0.78).

**Conclusion:**

The time spent on the smartphone by young adults does not influence the time to complete the GPT, indicating that smartphone use does not influence measures of manual dexterity.

**Supplementary Information:**

The online version contains supplementary material available at 10.1186/s12868-021-00639-y.

## Background

Manual dexterity is an important aspect of everyday life and it is associated with executive functions [1]. It is defined as the ability to manipulate objects and it is commonly evaluated through the time spent to complete the pegboard test [2]. There are two widely adopted pegboard tests, the 9-Hole Peg Test (9-HPT) and the Grooved Pegboard Test (GPT), which are both included in the National Institutes of Health (NIH) Toolbox for the Assessment of Neurological and Behavioural Function [3]. The GPT is also included in several neuropsychological batteries [4]. A key difference in time to complete the GPT is participant age, validating the reason this test is an appropriate method to evaluate hand function across the lifespan [5]. Unfortunately, the GPT seems influenced by different factors such as biological sex (females are usually faster than males) [4, 6, 7], education (people with higher education result to be faster) [6, 8], cognitive functions (cognitive tasks are more challenging than motor tasks) [9] and mental fatigue [10].

In the literature exists normative data, these are provided in peer-reviewed papers [2, 6, 7] and in the GPT user instructions (Lafayette Instruments, USA), with the most recent work on “time to completion” published in 2011 by Wang and colleagues [2]. Thus, the ever-increasing frequency of mobile device use, specifically smartphones and related mobile applications (e.g., games, maps, calculator, music, translators…), and social media (e.g., Facebook, Twitter, YouTube, Instagram, TikTok…) surely have an impact on daily life [11] and must be considered in evaluating manual dexterity. For example, the use of smartphones has been found to be the cause of a shorter time response on learning tasks and a less accurate working memory [12]. Working memory performance is also influenced by social media such as Facebook and YouTube [13] and it seems strongly associated with the time to conclude the GPT [9]. Another aspect to consider about new technologies is media multitasking, which is the control of more media or activity concurrently [14]. Media multitasking has been reported to impact neural structures by reducing the volume of the anterior cingulate cortex [14] and it is negatively related to cognitive control [15].

Because the GPT is adopted to evaluate neurological and behavioural functions [3], and considering that young adults spend more time on smartphones compared to older adults [16], it should be necessary to update the normative data related to adolescents and young adults. Before investing money to update the normative data for all the populations, a study in a specific population is required. Due to the decline of manual dexterity with aging [17] and the stabilization of the results only in early adolescence [18], the present investigation was designed to involve young adults. For this reason, the purpose of our study was to evaluate how time spent on a smartphone impacts GPT execution in young adults.

## Methods

### Participants

Young adults (19–24 years old) attending the University of Palermo and the Lithuanian Sports University were included for investigation to avoid that cultural and education level were possible confounding factors [19]. Instead, participants were excluded if: (i) they presented injuries or physical problems in their upper limbs such as fractures, medical interventions, prostheses, inflammation of the fingers, arms, or shoulder; (ii) they presented neurological diseases such as intellectual disability. Participants were recruited through social networks and flyers for this study. Each participant, before the study, was informed about the testing procedure, the benefits and the risks. All participants provided their written consent to take part in this research and allowed the use of their data. Participants were not financially compensated. The study was carried out in accordance with the ethical standards of the Declaration of Helsinki and it was approved by the Bioethics Committee of the University of Palermo (ID: 19/2020).

A total of 41 (25 women and 16 men) participants were included; a power analysis with G*Power software (version 3.1.9) at 0.80 revealed a minimum sample required of 26 participants. The mean age (standard deviation) was 20.7 (1.5) years, the height was 169.2 (28.7) cm, while the weight was 69.3 (13.9) kg (Table 1).

**Table 1:** Overview of the samples characteristics

	Age (years)	Height (cm)	Weight (kg)	t SM (h/week)	t GPT (s)	Right handed	Left handed
Overall	20.7 (1.5)	169.2 (28.7)	69.3 (13.9)	31.6 (12.8)	60.0 (6.9)	33	8
Women	20.9 (1.3)	167.0 (5.4)	60.3 (6.3)	31.3 (13.3)	58.9 (7.3)	21	4
Men	20.4 (1.8)	172.7 (46.2)	83.3 (10.3)	32.0 (12.6)	61.8 (6.0)	12	4

Data are expressed as means (standard deviation)

*GPT* grooved pegboard test, *SM* smartphone, *t* time

### Study design

Participants completed a single session lasting about 30 min. The session comprised the completion of a questionnaire and one GPT repetition. The questionnaire was made of questions regarding personal characteristics (age, gender, height, weight, handedness) and weekly time spent on smartphones or mobile devices (see Additional file 1). The Edinburgh Handedness Inventory (Short Form) was adopted to determine and define the dominance of the upper limbs [20]. Questions related to the time spent in the last seven days on the smartphone and the application or the most used social media were asked. The time spent in a week with the smartphone was assessed through the phone itself, through the settings each smartphone provides with the software iOS, and through the appropriate application if the smartphone had an Android operating system.

The GPT followed a procedure validated previously [2, 21] which consisted of placing pegs into twenty-five keyhole-shaped slots (in a 5-by 5 grid, with different keyhole orientations). The pegs were placed with one hand, one by one, while the other hand was on the desk. The test was performed from top to bottom and from left to right for right-handers while from right to left for left-handers. Participants filled the holes line by line, as quickly as possible. The GPT roles were described before and the participant familiarized with the test filling only the first top row. After the pegs were removed by the investigator, the participant was free to choose when to start the test. Time was measured from the moment the first peg touched the board to the moment the last peg was inserted. The time was recorded through a stopwatch.

### Statistical analysis

The statistical analysis was performed via GraphPad Prism 8.0 (San Diego, California, USA). The normality of the data was evaluated through the Shapiro–Wilks test with α set at 0.05. Differences between gender were evaluated through the *t*-test, if the data will be normally distributed. The correlation between the time to conclude the GPT and the time spent with the smartphone has been tested through a Pearson correlation analysis. The p value was set a 0.05.

## Results

**Fig. 1:**
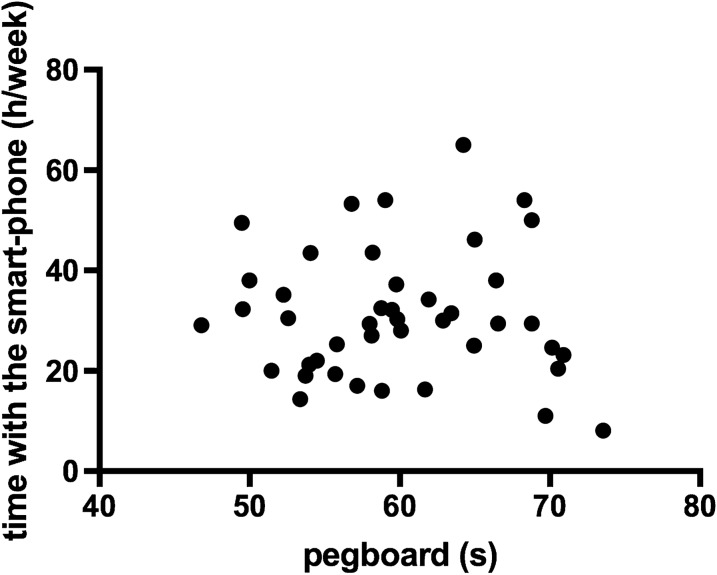
Correlation between GPT (s) and weekly time spent on a smartphone (h/week) (p = 0.78). *s* seconds, *h* hours

Data regarding the time spent on the smartphone and the GPT were normally distributed. With the Edinburgh Handedness Inventory (Short Form) it was possible to assess hand dominance: 33 participants were right-handed; the remainder were left-hand dominant. Notwithstanding no significant difference was observed across genders (p = 0.87), men, in general, spent more time on the smartphone compared to women [32.0 (12.6) vs 31.3 (13.2) h/week, for man and woman, respectively]. Most of the participants used mainly Facebook (51%) followed by Instagram (37%), YouTube (10%) and Snapchat (2%). No statistical difference was observed regarding the GPT execution time [58.9 (7.3) vs (61.8 (6.0) s, p = 0.20, for woman and man, respectively]. The correlation test for the GPT and the time spent using the smartphone is not significant (r = 0.044, p = 0.78) (Fig. 1). A summary of the values of statistics is presented in Table 2.

**Table 2:** Summary of the various values of statistics

Parameter examined	Statistical significance	p values
Women vs men	No	p = 0.87
GPT execution time	No	p = 0.20
GPT execution time vs smartphone time	No	p = 0.78

*GPT* grooved pegboard test

## Discussion

The results of the present study suggest that the time spent on the smartphone does not influence the time adopted to complete the GPT in young adults and consequently, it is not related to manual dexterity. These results do not support the initial hypothesis of our investigation. The study, consequently, indicates that the normative data proposed by Wang and colleagues [2] are still valid.

The time spent to complete the GPT in the present study [60.0 (6.9) s], in comparison with the normative data for young adults (21–30 years) proposed by Wang and colleagues [2] [55.3 (7.3) s], resulted higher of more than five seconds. Similar normative data to our study are the values proposed by Bornstein in 1985 [7] for young adults [20–39 years; GPT time: 60.9 (16.2) s] and by Ruff and Parker [6] [16–39 years; GPT time: 62.5 (9.6) s]. Furthermore, the present study confirms the results previously obtained in other studies [4, 6, 7] in which young women resulted faster than young men.

There is a lack of association between the time spent on the smartphone and the time required to complete the GPT, this finding could be explained by the different cerebellar activation zone that motor skills and cognition have, in young adults [22, 23]. The literature on this topic is contradictory with Cain and colleagues [24] that suggest media multitasking is related to executive functions and dexterity. Executive functions are strongly correlated with prehensile movements such as grasping, rotational speed of hand movements, and end-point movement speed, and consequently with the pegboard test [25]. While Inal and colleagues [26], instead, suggested that an inverse correlation exists between hand function and the time spent on the smartphone. The confusion regarding this topic is confirmed by the present study highlighting the necessity of a deeper investigation in which cognitive function, manual dexterity, and motor unit activation should be associated with time spent on smartphones.

The findings of this study want to help the community by providing updated information on the time to complete the GPT and feedback for future studies. This study presents some limits such as the sample recruited which was composed only by young adults and not adolescents, adults or older adults. Adolescents nowadays have been growing up and are very frequently with smartphones in their hands, and such could heavily influence manual dexterity. Since 2007 the Apple iPhone was introduced in the market, a device able to replace computers and laptops [16] and this has to be taken into account when comparing adolescents to other populations. Older adults, indeed, could be more influenced by their working and cultural background than the time spent on smartphones. Adults’ time to complete the GPT, instead, could be influenced by the typology of work performed, in this case, a distinction could be performed between a more oriented mechanic or technology work. Future studies, consequently, should have to compare the time to complete the GPT and the smartphone use in different population. Another limit of the study is the lack of cognitive tests to evaluate the influence of the smartphone on the cognitive system and to compare these results to the GPT time. One last limitation of the study was the approximate identification of the total time spent on the smartphones, consequently, future studies should have to consider its use in years and not only in the last week.

## Conclusions

Time spent on the smartphone does not relate to time to complete the GPT. Consequently, the time spent using a smartphone does not relate to manual dexterity in young adults and should not be considered a confounding variable on GPT completion in young men and women.

## Supplementary Information

**Additional file1.**

## Data Availability

The datasets used and/or analysed during the current study are available from the corresponding author on reasonable request.

## Abbreviations

GPT: Grooved Pegboard Test
9-HPT: 9-Hole Peg Test
NIH: National Institutes of Health
