# Supplementary material for: Time spent on the smartphone does not relate to manual dexterity in young adults
Source: BMC Neurosci. 2021 May 11;22:34. doi: 10.1186/s12868-021-00639-y (PMC8111913; doi:10.1186/s12868-021-00639-y)
Supplement: Supplementary file 1 — Additional file1. [file 12868_2021_639_MOESM1_ESM.pdf]

Participant id number: ______

Age _____________ Gender: Male / Female

Height (cm) __________ Weight (kg) _______

Number of hours you use your mobile phone in the last 7 days __________

Edinburgh Dominant Hand Determination Questionnaire

Specify the hand you use most often to perform the following actions or use the following items:

	Always right	Usually right	Both equally	Usually left	Always left
Writing					
Throwing					
Toothbrush					
Spoon					
